# Self-Nitrogen-Guided Activation of Algae Biomass into Hierarchical Porous Carbon Electrodes for Aqueous Supercapacitors

**DOI:** 10.3390/molecules31132329

**Published:** 2026-07-02

**Authors:** Wanxi Wang, Yuchen Tian, Haibin Li, Huan Liu

**Affiliations:** 1 College of Chemistry and Chemical Engineering, Hexi University, Zhangye 734000, China; wwx2026520@163.com; 2 School of New Materials Science and Chemical Engineering, Beijing Institute of Petrochemical Technology, Beijing 102617, China; 2024520299@bipt.edu.cn; 3 Key Laboratory of Energy Conversion Optoelectronic Functional Materials of Jiangxi Education Institutes, School of Mathematics and Physics, Jinggangshan University, Ji’an 343009, China

**Keywords:** algae biomass, self-nitrogen doping, hierarchical porous carbon, KOH activation, supercapacitor, pore engineering, electrochemical energy storage

## Abstract

Biomass-derived porous carbons are promising supercapacitor electrodes, but their electrochemical performance is often limited by the trade-off between activation-induced pore formation and heteroatom retention. In this work, algae biomass was used as an intrinsic N/O/S-containing precursor to prepare self-nitrogen-doped hierarchical porous carbon by pre-carbonization followed by controlled KOH activation. A temperature-, dosage- and time-dependent sample library was constructed to correlate activation conditions with textural properties, nitrogen configuration, wettability, charge-transfer resistance and electrochemical behavior. The optimized AHPC-850 sample exhibits a BET surface area of 1486 m^2^ g^−1^, a total pore volume of 0.96 cm^3^ g^−1^, a retained surface nitrogen content of 2.91 at.%, and a charge-transfer resistance of 0.41Ω. In a three-electrode configuration, AHPC-850 delivers 386 F g^−1^ at 1 A g^−1^ and retains 62.4% of its capacitance at 20 A g^−1^. During 10,000 cycles at 10 A g^−1^, the electrode maintains 96.3% capacitance retention with a stable coulombic efficiency above 98.8%. A symmetric aqueous device based on AHPC-850 achieves an energy density of 24.8 Wh kg^−1^ at 250 W kg^−1^. These results indicate that algae-derived carbon can be improved by balancing pore accessibility, nitrogen retention and transport resistance rather than by maximizing surface area alone.

## 1. Introduction

The increasing use of renewable electricity, portable electronics, intelligent sensing systems and electric transportation has intensified the demand for electrochemical energy-storage devices that combine high power output, long service life and operational safety [1,2,3]. Supercapacitors are attractive because they store and release charge rapidly and usually display better cycling stability than batteries [4,5,6]. Their relatively limited energy density, however, still restricts broader application. Since the energy density of a supercapacitor depends on the electrode capacitance and operating voltage, the development of electrode materials with accessible charge-storage sites and efficient ion/electron transport pathways remains essential.

Porous carbon materials are widely used as supercapacitor electrodes because of their tunable pore structure, chemical stability, moderate cost and electrical conductivity [7,8,9]. In carbon electrodes, micropores mainly provide interfacial area for electrical double-layer formation, while mesopores and macropores shorten ion-transport pathways and improve electrolyte penetration. Recent studies on hierarchical porous carbons further emphasize that interconnected pores, rather than surface area alone, are critical for energy-storage performance [10,11,12]. Nevertheless, excessive activation can damage conductive domains, generate inaccessible ultramicropores or remove heteroatom-containing functional groups; therefore, pore development must be balanced with surface chemistry and electronic continuity [13,14,15,16,17].

Biomass-derived carbon provides a sustainable route to porous electrode materials because biomass is renewable, abundant and often structurally heterogeneous [8,18,19]. However, many biomass carbons require severe chemical activation or external heteroatom doping to obtain sufficient capacitance, which can increase processing complexity and weaken the environmental advantage of the biomass route [20,21,22,23,24]. Algae biomass is particularly interesting because proteins, pigments, polysaccharides, lipids and mineral components provide intrinsic nitrogen, oxygen and sulfur sources as well as a chemically diverse carbon skeleton [22,25,26]. Algae-derived and microalgae-derived carbons have been reported for supercapacitors [22,26,27], but the relationship between activation severity, nitrogen retention, pore connectivity and device-level performance still requires clearer analysis.

Herein, algae-derived hierarchical porous carbon (AHPC) was prepared through pre-carbonization and KOH activation. The work focuses on the following question: how can activation conditions be selected to generate ion-accessible pores while retaining useful nitrogen species and maintaining low transport resistance? To address this question, non-activated carbon, temperature-dependent activated carbons, KOH-ratio-dependent carbons and activation-time-dependent carbons were prepared and compared. The samples were characterized by precursor analysis, nitrogen adsorption–desorption, pore-size distribution, XRD, Raman spectroscopy, XPS, wettability and electrolyte-uptake measurements, conductivity tests and electrochemical evaluation in both three-electrode and symmetric two-electrode configurations.

The results identify AHPC-850 as the optimized sample under the present experimental conditions. Its performance is discussed cautiously as the outcome of coupled textural, chemical and interfacial factors, including accessible micro/mesoporosity, retained nitrogen species, electrolyte affinity and low charge-transfer resistance. The study also separates three-electrode material behavior from symmetric-device performance and discusses practical challenges associated with algae variability, KOH activation, high-loading electrodes and scale-up.

## 2. Materials and Methods

### 2.1. Materials and Biomass Pretreatment

Dried green algae biomass was used as the renewable carbon precursor. Potassium hydroxide (KOH), hydrochloric acid (HCl), ethanol, polyvinylidene fluoride (PVDF), acetylene black, N-methyl-2-pyrrolidone (NMP), nickel foam, platinum plate, sodium sulfate (Na_2_SO_4_) and potassium hydroxide electrolyte were used as-received. Deionized water was used throughout all washing and filtration processes. Before carbonization, the algae biomass was washed several times with deionized water to remove soluble salts, sand particles and loosely attached impurities. The cleaned biomass was dried at 80 °C for 12 h, pulverized and sieved through a 100-mesh screen to improve batch homogeneity.

To minimize variability caused by precursor heterogeneity, the dried algae powder was mixed thoroughly before use, and each batch of precursor was divided into aliquots for parallel synthesis. Three independently pretreated precursor batches were processed under the same washing, drying, sieving, impregnation and post-treatment procedures to improve batch-to-batch consistency. This procedure is important for marine and freshwater biomasses because inorganic salts, proteins and polysaccharides may not be uniformly distributed.

The pretreatment step was deliberately kept mild. Strong acid or alkali pretreatment before carbonization can remove minerals and change the biochemical composition of algae, but it can also eliminate heteroatom-containing components that are valuable for self-doping. Therefore, only water washing was used before pre-carbonization, while acid washing was reserved for the post-activation stage to remove residual potassium and ash. This sequence helps preserve the intrinsic nitrogen-containing components during the early stage of carbon formation.

The precursor was characterized before carbonization because the chemical composition of biomass strongly affects carbon yield, heteroatom retention and pore development. Moisture, volatile matter, fixed carbon and ash content were estimated by proximate analysis. Elemental composition was evaluated by CHNS/O analysis. Fourier transform infrared spectroscopy (FTIR) was used to identify functional groups such as hydroxyl, amino, carbonyl and polysaccharide-related C–O species. Thermogravimetric analysis (TGA) was performed under nitrogen from room temperature to 900 °C to identify the main decomposition stages. These precursor-level measurements were used to justify the selection of algae as a self-doping carbon source and to determine the pre-carbonization temperature.

The relatively high nitrogen content in the algae precursor is essential for the self-doping concept. In conventional lignocellulosic biomass, nitrogen is usually scarce, and external nitrogen sources are often introduced to improve wettability and pseudocapacitance. In contrast, protein- and pigment-containing algae can provide nitrogen internally. However, high precursor nitrogen does not automatically produce a high-performance carbon electrode, because nitrogen species may be volatilized or buried inside inaccessible domains during carbonization. This is why the synthesis strategy focuses on the activation window rather than simply choosing a nitrogen-rich biomass.

### 2.2. Carbon Preparation and Activation Optimization

**Figure 1:**
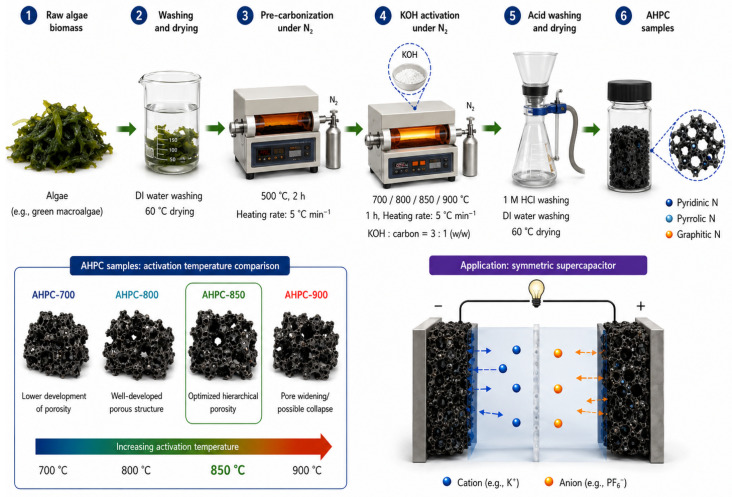
Experimental design and synthesis route for algae-derived nitrogen-doped hierarchical porous carbon (AHPC). The arrows indicate the chronological synthesis sequence, including raw algae pretreatment, pre-carbonization under N_2_, KOH-assisted activation, acid washing, drying and final electrode application. The lower panels summarize the activation-temperature sample library using irregular interconnected porous carbon blocks rather than idealized irregular porous carbon blocks, highlighting AHPC-850 as the optimized sample under the tested conditions because it balances pore generation, nitrogen retention and electrochemical capacitance.

The synthetic strategy is summarized in Figure 1. In a typical preparation, dried algae powder was placed in an alumina boat and pre-carbonized at 500 °C for 2 h under flowing nitrogen at a heating rate of 5 °Cmin^−1^. The purpose of pre-carbonization was to remove unstable volatiles, stabilize the carbonaceous skeleton and partially transform algae-derived nitrogen-containing biopolymers into carbon-bound nitrogen species. After cooling to room temperature, the pre-carbonized material was ground into a fine powder and denoted as algae char.

The two-step thermal process was selected instead of direct one-step activation for two reasons. First, direct mixing of raw biomass with KOH can cause violent gas release, uneven activation and poor reproducibility because raw algae contains moisture, proteins and volatile compounds. Second, pre-carbonization produces a more stable char that can be impregnated more uniformly with KOH. The pre-carbonization temperature of 500 °C was chosen according to the TGA profile, where the major decomposition of algae biopolymers is substantially completed while the carbon skeleton still retains chemically reactive sites for subsequent activation.

The KOH activation mechanism is generally associated with dehydration, redox etching and intercalation reactions. At elevated temperature, KOH and its decomposition products react with carbon, generating K-containing compounds, H_2_, CO and CO_2_, which open the carbon framework. Metallic potassium may also intercalate into carbon layers and expand the structure. After acid washing, these inorganic species are removed, leaving behind a porous carbon matrix. In the algae-derived system, this activation process occurs simultaneously with nitrogen transformation. Thus, the activation temperature must be high enough to create pores but not so high that nitrogen functionalities are completely eliminated.

For chemical activation, the algae char was mixed with KOH at different char:KOH mass ratios. Unless otherwise stated, the optimized ratio was 1:3. The mixture was homogenized with a small amount of deionized water, stirred for 4 h, dried at 100 °C and then activated under flowing nitrogen. Temperature-dependent samples were prepared at 700 °C, 800 °C, 850 °C and 900 °C for 1.5 h; they were denoted as AHPC-700, AHPC-800, AHPC-850 and AHPC-900, respectively. A non-activated control sample was obtained by heating the algae char at 850 °C without KOH and was denoted as AC-850.

After activation, the black products were washed with 1 M HCl to remove potassium salts, residual inorganic species and ash-derived residues. The acid-washed products were repeatedly rinsed with deionized water until the filtrate reached nearly neutral pH. Finally, the products were dried at 80 °C overnight. The carbon yield was calculated based on the mass ratio between the final dried carbon and the initial pre-carbonized char.

To make the experimental design systematic, three activation variables were considered. First, activation temperature was varied to investigate the competition between pore generation and nitrogen retention. Second, the char:KOH mass ratio was varied from 1:1 to 1:4 to determine whether stronger chemical etching improved or weakened the electrochemical output. Third, activation time was varied from 0.5 h to 2.5 h at 850 °C to evaluate the time window of the activation reaction. An integrated performance score was calculated by normalizing the BET surface area, pore volume, inverse charge-transfer resistance and capacitance while penalizing severe nitrogen loss. This score was used only to guide experimental selection and did not replace direct electrochemical evaluation.

The use of three optimization dimensions is important because activation temperature, KOH dosage and holding time are not interchangeable. Temperature determines the thermodynamic severity of carbon etching and nitrogen volatilization. KOH dosage controls the number of chemically reactive etching sites and therefore affects micropore formation, pore widening and carbon yield. Activation time controls how far the etching reaction proceeds after the target temperature is reached. A material obtained at high temperature for a short time may not be equivalent to a material obtained at lower temperature for a longer time, because nitrogen transformation, graphitic-domain rearrangement and inorganic-species removal occur through different kinetic pathways. Therefore, the experimental matrix was designed to avoid overinterpreting a single synthesis parameter.

For each optimization series, the final carbon was evaluated using yield, BET surface area, total pore volume, nitrogen content, $R_{ct}$ and capacitance at 1 Ag^−1^. The preferred condition was selected only when improvements in capacitance were accompanied by reasonable rate capability and cycling stability. This criterion prevents the selection of a highly activated sample that gives high low-rate capacitance but poor durability or excessive loss of yield. In practical biomass conversion, yield and washing burden are also important, because a material with excellent laboratory performance may be unattractive if it requires very severe activation and produces substantial alkaline waste.

### 2.3. Material Characterization and Interfacial Evaluation

Surface morphology was examined by field-emission scanning electron microscopy (FE-SEM) at an accelerating voltage of 5–10 kV after the samples were fixed on conductive carbon tape and sputtered with a thin Au layer when required. Transmission electron microscopy (TEM) and high-resolution TEM were performed at 200 kV to observe the local pore-wall texture and graphitic fringes. Images were collected from multiple regions of each sample to avoid interpreting isolated local features.

Nitrogen adsorption–desorption measurements were conducted at 77 K. Before analysis, the carbon samples were degassed at 200 °C under vacuum for at least 10 h. The Brunauer–Emmett–Teller (BET) surface area was calculated from the linear region satisfying the BET consistency criteria, the total pore volume was estimated from the adsorption quantity at $P/P_{0}\approx 0.99$, and the pore-size distribution was obtained using a nonlocal density functional theory (NLDFT) model. The isotherms, BET surface area and pore-size distribution were interpreted together because pore accessibility and pore connectivity are more relevant to supercapacitor behavior than a single surface-area value [16,17].

X-ray diffraction (XRD) patterns were recorded with Cu Kα radiation (λ=0.15406 nm) over the range of 10–80° at a scan rate of 5° min^−1^. Raman spectra were collected using a 532 nm laser, and the $I_{D}/I_{G}$ ratio was obtained after baseline correction. The D band near 1350 cm^−1^ reflects defect/disordered carbon, while the G band near 1580 cm^−1^ corresponds to graphitic sp^2^ carbon domains. Therefore, the $I_{D}/I_{G}$ ratio was used as a comparative indicator of disorder/graphitization rather than as an independent measure of electrochemical performance [28].

X-ray photoelectron spectroscopy (XPS) was performed using Al Kα radiation, and all binding energies were calibrated with the C 1 s peak at 284.8 eV. For N 1 s fitting, a Shirley background was subtracted and mixed Gaussian–Lorentzian functions were used. The peak positions and full width at half maximum (FWHM) values were constrained within commonly accepted ranges for pyridinic N, pyrrolic N, graphitic N and oxidized N. The fitted residual was inspected to avoid overfitting. Because XPS is surface-sensitive, the N 1 s results are interpreted as surface nitrogen speciation; they do not necessarily represent the full bulk nitrogen distribution of the carbon particles.

Water contact-angle measurements were used only as an initial indicator of surface polarity. To address the difference between pure water and operating electrolytes, electrolyte-uptake tests were carried out using the same aqueous electrolytes used for electrochemical testing. Dried electrodes were immersed for a fixed period, surface liquid was removed, and the mass increase was recorded. Four-point probe conductivity measurements were performed on compressed carbon pellets, and the results were interpreted together with EIS data to evaluate electronic and interfacial transport.

### 2.4. Electrode Fabrication, Electrochemical Testing, and Data Reliability

Working electrodes were prepared by mixing the active carbon material, acetylene black and PVDF binder at a mass ratio of 8:1:1 in NMP. The slurry was stirred for 6 h until a uniform dispersion was obtained, coated onto cleaned nickel foam and dried at 80 °C. The electrode was then pressed gently to improve contact between the active layer and the current collector. Unless otherwise noted, the standard active-material mass loading was controlled at 2.4±0.2 mg cm^−2^. For loading-dependent tests, electrodes with mass loadings of 1.2, 2.4, 4.8 and 7.5 mg cm^−2^ were fabricated.

Before electrochemical testing, the electrodes were immersed in the electrolyte under vacuum for a short period to improve wetting of the inner pores. This procedure is particularly important for high-surface-area carbons because trapped air in micropores and mesopores may lead to underestimated capacitance and unstable initial cycling. The geometric area of each electrode was kept constant, and the active-material mass was measured after drying. The mass of nickel foam and binder was not included in the gravimetric capacitance calculation, following common practice in material-level electrode evaluation. However, the two-electrode device calculations were based on the total active mass of both electrodes.

To evaluate the optimized material beyond a single low-loading test, additional electrodes with higher mass loading were prepared. High mass loading normally increases ion-diffusion length and reduces utilization of internal surface area. Therefore, mass-loading tolerance provides useful evidence that the hierarchical pore structure is not only beneficial in thin laboratory electrodes but also relevant to more practical electrode configurations.

Three-electrode measurements were conducted in 6 M KOH electrolyte using the prepared carbon electrode as the working electrode, a platinum plate as the counter electrode and a Ag/AgCl electrode as the reference electrode. The potential window was set from −1.0 to 0 V vs. Ag/AgCl unless otherwise stated. Cyclic voltammetry (CV) was performed at scan rates from 5 to 200 mV s^−1^. Galvanostatic charge–discharge (GCD) curves were collected at current densities from 0.5 to 50 A g^−1^, and the discharge time was taken from the linear discharge branch after excluding the IR drop. Electrochemical impedance spectroscopy (EIS) was performed from 100 kHz to 10 mHz with an amplitude of 5 mV. Long-term cycling tests were conducted at 10 A g^−1^, and coulombic efficiency was calculated from the ratio of discharge time to charge time in each cycle.

The gravimetric capacitance of a single electrode was calculated from GCD curves according to

(1) $$C=\frac{I\Delta t}{m\Delta V},$$

where *C* is the specific capacitance, *I* is the discharge current, Δt is the discharge time, *m* is the active mass of the carbon material only, and ΔV is the effective potential window after excluding the IR drop. The masses of nickel foam, binder and conductive additive were not included in *m*. Kinetic analysis was conducted using the power-law relationship

(2) $$i=av^{b},$$

where *i* is the peak current, *v* is the scan rate, and the *b* value indicates whether the process is diffusion-controlled or surface-controlled. The capacitive contribution was further estimated using $i\left(V\right)=k_{1}v+k_{2}v^{1/2}$ at selected potentials.

#### Symmetric Supercapacitor Assembly and Device Evaluation

Symmetric supercapacitors were assembled using two AHPC-850 electrodes with balanced active-material masses, a porous separator and 1 M Na_2_SO_4_ aqueous electrolyte. The mass difference between the two electrodes was controlled below 5%, and the total active mass of both electrodes was used for device-level energy and power calculations. The stable operating voltage window was evaluated from 1.0 to 1.8 V and fixed at 1.6 V for device-performance comparison. Device CV curves, GCD curves and EIS spectra were collected under the same electrochemical workstation settings used for three-electrode tests. For the Nyquist plots, the x-axis and y-axis were displayed on the same numerical scale to avoid distortion of impedance features [29]. The device capacitance was calculated by

(3) $$C_{cell}=\frac{I\Delta t}{M\Delta V},$$

where *M* is the total active mass of both electrodes. Energy density (Wh kg^−1^) and power density (W kg^−1^) were calculated as

(4) $$E=\frac{C_{cell}V^{2}}{7.2},$$

(5) $$P=\frac{3600E}{\Delta t}.$$

Self-discharge, cycling durability and mass-loading tolerance were evaluated to assess practical applicability.

Because high surface area alone does not guarantee fast electrolyte access, additional interfacial measurements were included in the experimental design. Static water contact-angle measurements were used as a first indicator of surface wettability. Although a water droplet is not identical to a concentrated KOH electrolyte, a lower contact angle generally indicates improved surface polarity and easier electrolyte penetration. Electrolyte uptake was measured by immersing dried electrodes in electrolyte for a fixed period, removing surface liquid and recording the mass increase. This measurement provides a practical estimate of how readily the electrode absorbs electrolyte within its porous network.

Four-point probe conductivity measurements were performed because electronic transport is an important but often overlooked factor in porous carbon electrodes. Excessive activation can improve surface area while weakening the carbon framework and increasing resistance. Therefore, conductivity was evaluated together with porosity and nitrogen content to distinguish whether high-rate performance originates from intrinsic carbon properties or from electrode-fabrication effects.

The electrode-fabrication parameters were deliberately kept constant across samples so that differences in electrochemical performance could be attributed primarily to the carbon materials. The active-material loading, binder ratio, conductive carbon ratio and drying protocol were maintained for all standard electrodes, and the additional loading-dependent electrodes were used to examine ion-transport limitations at larger electrode thicknesses.

Control experiments were incorporated at three levels. First, AC-850 was used as a non-activated carbon control to evaluate whether thermal carbonization alone could generate useful porosity. Second, temperature-dependent AHPC samples were compared to determine whether the optimized performance was caused by a genuine balance between porosity and nitrogen retention rather than by a random synthesis condition. Third, the symmetric device was assembled to verify whether three-electrode performance translated into a practical two-electrode configuration. These controls are important because three-electrode measurements often overestimate the practical performance of carbon electrodes.

To support reproducibility, each electrochemical test was performed using at least three independently prepared electrodes from the same carbon batch, and the reported values represent reproducible averages. Long-term cycling was verified with independently assembled cells. SEM/TEM images were selected from multiple observed regions, and XPS spectra were analyzed using consistent fitting constraints. The BET adsorption–desorption data, XPS survey and fitting outputs, CV/GCD traces, EIS spectra and cycling records were obtained from the original instrument files and retained by the authors for data verification.

## 3. Results and Discussion

### 3.1. Synthesis Optimization and Multi-Descriptor Material Selection

The suitability of algae as a carbon precursor originates from its biochemical composition. Compared with woody biomass, algae generally contains a larger fraction of proteins and pigments, which can serve as intrinsic nitrogen reservoirs during carbonization. The measured precursor data in Table 1 indicate that the algae feedstock contains appreciable nitrogen and oxygen, together with mineral ash that must be removed after activation. This composition explains why the synthesis route was designed around self-doping and post-activation acid washing. From a top-down material-design viewpoint, the precursor is not treated as an inert carbon source; instead, its native chemical complexity is used as part of the functional design.

The experimental route shown in Figure 1 therefore has a clear logic. Washing and drying reduce extrinsic variability. Pre-carbonization stabilizes the biomass and converts labile biochemical components into a carbonaceous scaffold. KOH impregnation introduces a chemical etchant into the char. High-temperature activation opens the pore system and simultaneously transforms part of the algae-derived nitrogen into carbon-bound nitrogen species. Acid washing removes inorganic residues, leaving a porous carbon network. The sample library then allows the effects of activation severity to be separated from precursor chemistry. This structured synthesis plan links precursor composition, activation severity and electrochemical behavior more explicitly than a single-condition biomass carbonization procedure.

The optimization matrix in Table 2 was designed to answer three questions. First, what activation temperature maximizes electrochemically useful porosity without excessive nitrogen loss? Second, how much KOH is required to create hierarchical pores without unnecessary over-etching? Third, what activation duration completes pore development while retaining structural integrity? The following results show that AHPC-850 is optimal because it is located at the intersection of these three requirements, not because it maximizes any single parameter.

The activation-parameter matrix avoids an over-simplified conclusion based only on the best-performing sample. Table 3 summarizes the measured trends for activation temperature, KOH dosage and activation time. Increasing activation temperature from 700 °C to 850 °C improves surface area, pore volume and capacitance, but further increasing the temperature to 900 °C decreases the useful nitrogen content and slightly lowers capacitance. A similar non-monotonic trend is observed for KOH dosage. Insufficient KOH does not fully open the pore network, whereas excessive KOH causes pore widening, lower yield and weaker structural integrity. Activation time also exhibits an optimum because short activation is incomplete, while prolonged activation continues to etch the carbon skeleton.

This optimization behavior shows that the selected sample is supported by a convergent trend across several independent descriptors. If the performance improvement was caused only by increasing surface area, the most strongly activated sample would be expected to perform best. If it was caused only by nitrogen content, the non-activated or mildly activated sample would be expected to perform best. Instead, AHPC-850 achieves the highest capacitance because it preserves enough nitrogen while forming a highly accessible micro/mesopore network. This non-monotonic trend is consistent with a mechanistic trade-off between pore generation and nitrogen retention.

The optimization table is interpreted as a design rule rather than as a simple list of values. The activation condition must be strong enough to expose charge-storage surface and create ion highways, but mild enough to avoid losing surface chemistry and carbon connectivity. This rule explains the behavior of the temperature series, the KOH-ratio series and the time series using the same mechanism, thereby strengthening the generality of the conclusion.

### 3.2. Structural, Chemical and Interfacial Characterization

**Figure 2:**
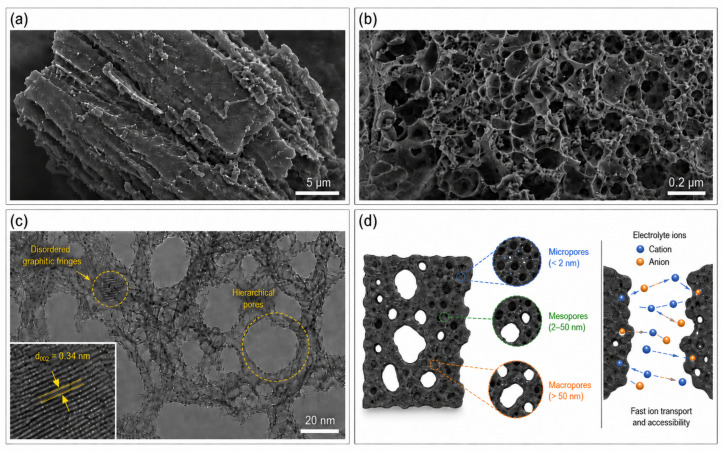
Morphology and microstructure characterization of algae-derived hierarchical porous carbon. (**a**) SEM morphology of raw algae-derived char showing a compact layered carbon structure. (**b**) SEM morphology of AHPC-850 showing an irregular interconnected porous network with rough pore walls and heterogeneous open channels after KOH activation. (**c**) TEM/HRTEM microstructure of AHPC-850, revealing sheet-like porous carbon domains, hierarchical voids and short-range disordered graphitic fringes. (**d**) Planar schematic illustration of the hierarchical pore architecture and ion-transport behavior, where micropores provide charge-storage sites and mesopores/macropores facilitate electrolyte penetration and ion migration.

The morphology and local microstructure of the algae-derived carbons are summarized in Figure 2. The raw algae-derived char exhibits a relatively compact and layered morphology, indicating that thermal carbonization alone is insufficient to generate an open pore network. Such compact morphology is unfavorable for supercapacitor electrodes because electrolyte ions cannot fully access the inner carbon domains. After KOH activation, the optimized AHPC-850 sample shows an irregular, sheet-like interconnected porous framework with rough pore walls, heterogeneous pore sizes and partially collapsed edges rather than an overly idealized ordered network. This morphological evolution shows that KOH activation transforms the dense algae-derived carbon skeleton into an ion-accessible porous carbon while preserving a defect-rich texture.

The TEM/HRTEM panels in Figure 2c further show that AHPC-850 contains sheet-like porous carbon domains with irregular voids and locally thin carbon walls. The local lattice fringes are short and distorted, which is characteristic of partially graphitized but defect-rich carbon. Such a structure is desirable for electrochemical capacitors: short-range graphitic domains can facilitate electron conduction, while defects, edges and nonuniform carbon walls can expose adsorption sites and improve electrolyte interaction. The planar ion-transport schematic in Figure 2d clarifies the intended hierarchical architecture by representing the material as a nonuniform sheet-like porous framework rather than an isolated ideal particle. Micropores (<2 nm) mainly contribute to electrical double-layer charge storage, mesopores (2–50 nm) provide ion-transport channels, and larger macropores reduce diffusion resistance by serving as electrolyte-buffering reservoirs.

The visual difference between dense char and AHPC-850 also supports the central synthetic hypothesis. The algae precursor already contains carbon, nitrogen, oxygen and sulfur-containing biopolymers, but those chemical advantages cannot be fully utilized unless the carbon skeleton is opened. Controlled KOH activation converts the biochemical precursor into an accessible carbon framework, while excessive activation must be avoided because it may destroy the conductive skeleton and remove nitrogen functionalities. Therefore, morphology is not merely descriptive; it provides direct evidence that the optimized sample combines open pore accessibility with structural continuity.

**Figure 3:**
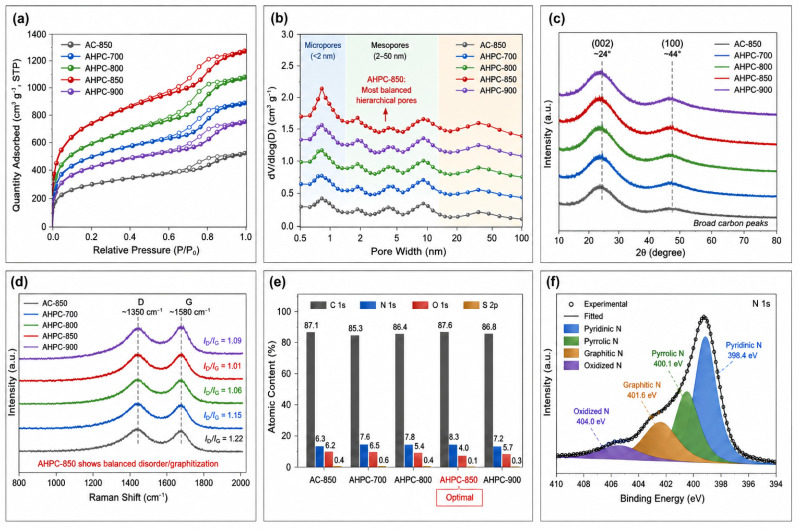
Structural and chemical characterization of algae-derived N-doped hierarchical porous carbon. (**a**) N_2_ adsorption–desorption isotherms of AC-850 and AHPC samples. (**b**) NLDFT pore-size distributions showing micropore and mesopore regions. (**c**) XRD patterns with broad turbostratic carbon reflections. (**d**) Raman spectra and $I_{D}/I_{G}$ ratios. (**e**) Surface elemental composition from XPS survey analysis. (**f**) High-resolution N 1 s XPS deconvolution of AHPC-850, showing pyridinic N, pyrrolic N, graphitic N and oxidized N species.

The pore-structure and chemical-state results are integrated in Figure 3. Nitrogen adsorption–desorption isotherms (Figure 3a) show a rapid uptake at low relative pressure, indicating the formation of micropores. The continuous adsorption increase and the hysteresis contribution at medium-to-high relative pressure suggest the presence of mesopores and larger transport channels. The NLDFT pore-size distributions (Figure 3b) further show that the activated samples contain both microporous and mesoporous domains. AHPC-850 does not simply maximize surface area; instead, it provides a comparatively balanced pore distribution, which is more relevant to ion-accessible capacitance than BET surface area alone [16,17].

The XRD patterns in Figure 3c exhibit broad peaks near 24° and 44°, corresponding to the (002) and (100) reflections of turbostratic carbon. The absence of sharp crystalline peaks suggests that the framework is mainly amorphous or weakly ordered, which is typical for activated biomass-derived carbon. Raman spectra (Figure 3d) show the D band and G band associated with disordered carbon and graphitic sp^2^ domains, respectively. The increase in $I_{D}/I_{G}$ after activation indicates that KOH etching introduces additional defects and edge sites while opening the pore structure. AHPC-850 shows an intermediate $I_{D}/I_{G}$ value: it contains sufficient defects to provide accessible adsorption sites, but it is not as severely etched as AHPC-900. This result supports the interpretation that pore development and structural disorder must be balanced rather than maximized [28].

The quantitative summary in Table 4 further supports this coupled-optimization interpretation. AHPC-850 shows the largest $S_{BET}$ and $V_{tot}$, a moderate nitrogen content, the lowest $R_{ct}$ and the highest capacitance among the prepared samples, whereas AHPC-900 indicates that excessive activation can reduce nitrogen retention and weaken electrochemical performance.

XPS analysis confirms the self-nitrogen-doping nature of the algae-derived carbon. As shown in Figure 3e, AHPC-850 retains surface nitrogen after high-temperature activation, demonstrating that algae-derived nitrogen-containing biopolymers can be transformed into carbon-bound nitrogen functionalities. The high-resolution N 1 s spectrum (Figure 3f) was fitted using constrained peak positions and FWHM values (Table 5). Pyridinic and pyrrolic nitrogen may improve surface polarity and provide redox-active edge sites, while graphitic nitrogen can facilitate charge transfer by modifying the carbon electronic structure. These assignments are used as supportive evidence rather than as direct quantification of the faradaic contribution, because XPS probes only the near-surface region and does not resolve the full bulk nitrogen distribution.

Figure 3 indicates that AHPC-850 is not optimized by one parameter alone. Although strong activation can increase porosity, it can simultaneously reduce nitrogen retention. Conversely, mild activation can preserve nitrogen but does not fully open the carbon skeleton. AHPC-850 represents a compromise state in which hierarchical porosity, defect density and surface nitrogen chemistry are synchronized. This structure–chemistry balance helps explain the electrochemical behavior beyond a simple BET surface-area argument.

The nitrogen-species distribution in Table 5 explains why total nitrogen content is insufficient as a sole descriptor. A high nitrogen concentration dominated by unstable oxidized species does not necessarily benefit cycling stability. Conversely, a moderate nitrogen content containing pyridinic, pyrrolic and graphitic components can improve wettability, provide redox-active surface sites and support conductivity. The interpretation is made cautiously because the N 1 s spectrum reflects surface chemistry, whereas the electrochemical response depends on the accessibility and stability of these sites within the porous electrode.

To further connect chemical structure with electrochemical behavior, interfacial properties were analyzed as summarized in Table 6. The contact angle decreases after activation and reaches the lowest value for AHPC-850, indicating improved wettability. This improvement is attributed to the combined effect of open pores and retained polar nitrogen/oxygen species. Electrolyte uptake follows a similar trend, showing that AHPC-850 can absorb electrolytes more efficiently than the non-activated control. The conductivity values indicate that activation improves transport initially, but overactivation does not further improve conductivity because excessive etching disrupts the framework.

These interfacial data bridge material characterization and electrochemical testing. BET analysis measures gas-accessible surface area under dry conditions, whereas electrochemical performance depends on electrolyte-accessible surface area under wet, charged conditions. Water contact angle alone cannot fully represent wetting in concentrated KOH or Na_2_SO_4_ electrolyte; therefore, contact-angle data were interpreted together with electrolyte uptake, EIS and rate behavior. AHPC-850 combines accessible porosity with improved electrolyte affinity, which contributes to its lower impedance and acceptable rate retention.

### 3.3. Three-Electrode Electrochemical Behavior and Symmetric-Device Performance

#### 3.3.1. Three-Electrode Performance

**Figure 4:**
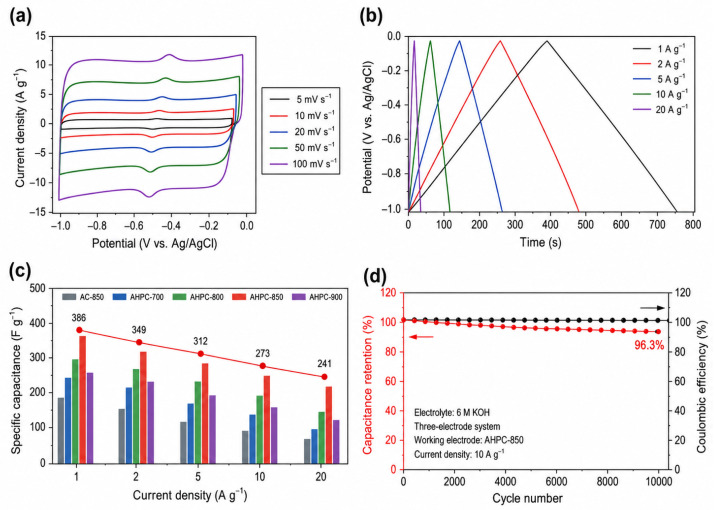
Three-electrode electrochemical performance of algae-derived N-doped hierarchical porous carbon. (**a**) CV curves of AHPC-850 at different scan rates. (**b**) GCD curves of AHPC-850 at different current densities. (**c**) Rate-capability comparison of AC-850 and AHPC samples; retention is defined as $C_{20\;A\;g^{-1}}/C_{1\;A\;g^{-1}}\times 100\%$. (**d**) Long-term cycling stability and coulombic efficiency of AHPC-850 at 10 A g^−1^.

The electrochemical behavior of AHPC-850 was first evaluated in a three-electrode configuration. Figure 4a presents the CV curves of AHPC-850 at different scan rates. The curves maintain a quasi-rectangular shape with weak redox humps, suggesting that the electrode stores charge through a dominant electrical double-layer mechanism with additional surface redox contribution from nitrogen/oxygen-containing groups. The preserved curve shape at high scan rates confirms rapid ion diffusion and good capacitive reversibility.

The GCD curves in Figure 4b show nearly symmetric triangular profiles, indicating reversible charge storage under the selected potential window. The slight deviation from an ideal triangle is consistent with a small surface redox contribution from nitrogen/oxygen-containing sites, but the faradaic contribution is not quantified solely from the CV shape. Based on the discharge branch after IR-drop correction, AHPC-850 delivers 386 F g^−1^ at 1 A g^−1^. This value was calculated using only the active carbon mass and the effective discharge window, as described in Section 2.3.

The rate-capability comparison in Figure 4c shows that AHPC-850 retains 62.4% of its capacitance when the current density increases from 1 A g^−1^ to 20 A g^−1^. This retention is acceptable for an algae-derived porous carbon and is discussed as competitive rather than exceptional, because recent biomass-derived carbons may display different retention values depending on current range, pore connectivity and electrode loading. The long-term cycling test (Figure 4d) shows 96.3% capacitance retention after 10,000 cycles. The coulombic efficiency remains above 98.8% during cycling, supporting good reversibility of the charge–discharge process.

The trend in Table 7 reinforces the concept that electrochemical performance is controlled by a coupled descriptor set. AC-850 retains more nitrogen but has limited porosity, resulting in low capacitance. AHPC-900 has strong activation but lower nitrogen retention and a less favorable balance between pore widening and conductive integrity. AHPC-850 shows the highest capacitance among the prepared samples because its accessible surface area, pore volume, nitrogen functionality and interfacial resistance are jointly balanced. This interpretation does not imply that pore hierarchy alone determines capacitance; instead, the electrochemical response is governed by the combined effects of pore accessibility, surface chemistry, conductivity and electrode wetting.

**Figure 5:**
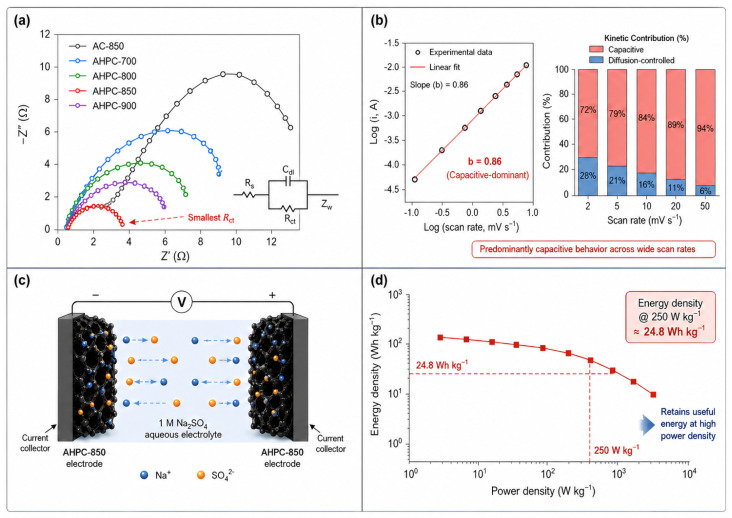
Kinetic analysis and symmetric-device evaluation of AHPC-850. (**a**) Nyquist plots of AC-850 and AHPC samples with identical x/y-axis scales. (**b**) Determination of the b value and capacitive contribution of AHPC-850. (**c**) Schematic illustration of the AHPC-850 symmetric device in 1 M Na_2_SO_4_ electrolyte. (**d**) Ragone plot of the AHPC-850 symmetric supercapacitor.

To further understand charge-transfer and ion-transport behavior, EIS and kinetic analyses were performed (Figure 5). The Nyquist plots in Figure 5a were displayed with identical x- and y-axis scales to avoid visual distortion of the impedance response. AHPC-850 exhibits the smallest high-frequency semicircle, corresponding to the lowest charge-transfer resistance. This result agrees with the structural and chemical characterization: connected pores reduce ion-diffusion barriers, while partially ordered carbon domains and graphitic nitrogen contribute to electron transport.

The kinetic relationship $i=av^{b}$ was used to estimate the dominant charge-storage process. A *b* value close to 1 indicates surface-controlled capacitive behavior, whereas a value close to 0.5 indicates diffusion-controlled behavior. As shown in Figure 5b, AHPC-850 exhibits a *b* value of approximately 0.86. The capacitive contribution increases from about 69.5% at 5 mV s^−1^ to 88.7% at 100 mV s^−1^, indicating that rapid surface-controlled storage dominates at higher scan rates. This kinetic separation supports the presence of fast interfacial charge storage but does not independently quantify the individual contribution of each nitrogen species.

#### 3.3.2. Symmetric Two-Electrode Device Performance

To evaluate practical applicability, a symmetric device was assembled using AHPC-850 as both electrodes. The device CV curves maintain a nearly rectangular profile within a 1.6 V window, and the GCD curves show quasi-triangular charge–discharge behavior, indicating reversible capacitive storage in the two-electrode configuration (Figure 5c,d). The device EIS spectrum (Figure 5e) shows a small high-frequency intercept and a steep low-frequency line, suggesting relatively low internal resistance and efficient ion transport. The Ragone plot in Figure 5f gives an energy density of 24.8 Wh kg^−1^ at 250 W kg^−1^, and useful energy output is retained at elevated power density. These two-electrode results are discussed separately from the three-electrode data because the device calculation is based on the total active mass of both electrodes and provides a more practical assessment of the material.

The device metrics in Table 8 show that AHPC-850 maintains useful energy output as power density increases. Many porous carbons show attractive low-rate capacitance but lose energy rapidly at high power because ions cannot access internal pores quickly enough. The present symmetric-cell results indicate that the pore/transport balance observed in the three-electrode tests is partly retained at device level, although further validation under higher mass loading and practical cell formats is still required.

### 3.4. Structure–Property–Performance Correlation and Literature Benchmarking

**Figure 6:**
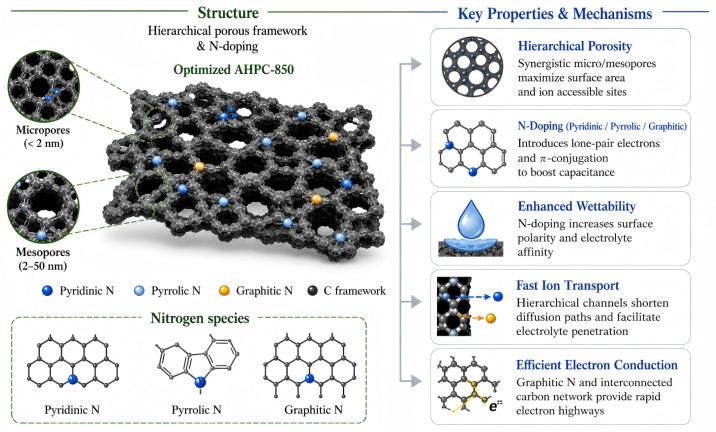
Structure–property relationship of AHPC-850. The schematic emphasizes the nonuniform interconnected porous framework and key nitrogen species; the arrows and color-coded regions indicate the coupled links among hierarchical porosity, nitrogen-induced surface functionality, enhanced wettability, fast ion transport and efficient electron conduction within the optimized algae-derived carbon.

The structure–property relationship of AHPC-850 is illustrated in Figure 6. The schematic focuses on the material-level origin of the electrochemical behavior rather than repeating a separate performance-summary block. Specifically, the irregular interconnected porous framework provides accessible micro/mesoporous channels, the retained pyridinic/pyrrolic/graphitic nitrogen species regulate surface polarity and interfacial charge storage, and the partially graphitized carbon framework supports electron conduction. These coupled structural features explain why AHPC-850 simultaneously exhibits strong electrolyte accessibility, rapid ion/electron transport and stable interfacial kinetics.

The mechanistic map in Figure 6 also summarizes why AHPC-850 performs better than the other samples under the tested conditions. AC-850 retains more nitrogen but lacks sufficient porosity. AHPC-700 and AHPC-800 show progressively improved pore structures but do not yet achieve the optimal balance. AHPC-900 is strongly activated, but the decrease in nitrogen content and possible over-etching reduce the useful electrochemical benefit. AHPC-850 is therefore not simply the sample with the highest activation severity; it is the sample in which pore generation and nitrogen retention are best synchronized.

The capacitance, energy density and power density discussed in this section were calculated using the equations and mass-normalization rules described in Section 2.4. In particular, single-electrode capacitance was obtained from the discharge branch of the GCD curves after excluding the IR drop, whereas the energy and power densities of the symmetric device were normalized to the total active mass of the two electrodes.

#### Benchmarking with Selected Biomass-Derived Carbon Electrodes

Table 9 compares the present work with selected biomass-derived carbon electrodes. Direct comparison requires caution because electrode mass loading, electrolyte, voltage window and testing configuration vary among reports; nevertheless, AHPC-850 shows competitive capacitance, rate capability and cycling durability. In addition, the strategy avoids additional nitrogen precursors by using the intrinsic nitrogen chemistry of algae biomass. This self-doping route reduces synthetic complexity and supports a more sustainable design philosophy.

The numerical comparison in Table 9 shows that AHPC-850 provides a competitive combination of capacitance, cycling stability and device-level energy output among biomass-derived porous carbons. Its rate retention of 62.4% from 1 to 20 A g^−1^ should be regarded as acceptable rather than exceptional. The comparison is not intended to rank materials directly, because reported values depend on testing configuration, electrolyte, voltage window, electrode loading and calculation basis. The main distinction of AHPC-850 is that competitive capacitance and stable cycling are achieved by using algae-derived nitrogen and controlled activation without adding an external nitrogen precursor.

### 3.5. Mechanistic Interpretation, Practical Applicability and Challenges

The results indicate that AHPC-850 represents an intermediate activation state rather than the most strongly etched carbon. Activation plays two competing roles: it opens the carbon framework and exposes the electrolyte-accessible surface, but excessive activation lowers yield, removes nitrogen species and can weaken conductive domains. The performance trend across the temperature, KOH ratio and time series therefore supports a balanced-activation interpretation. This conclusion is also consistent with the BET, Raman, XPS, EIS and electrochemical data: AHPC-850 combines accessible micro/mesoporosity, moderate structural disorder, retained surface nitrogen and relatively low charge-transfer resistance.

The role of pore hierarchy should nevertheless be interpreted together with surface chemistry and conductivity. Micropores provide interfacial area for double-layer charge storage, whereas mesopores and larger voids facilitate electrolyte penetration and reduce diffusion resistance. However, the capacitance cannot be predicted from pore structure alone. Nitrogen-containing sites may improve wettability and introduce redox-active surface centers, but their contribution depends on bonding configuration, accessibility and stability. The kinetic analysis indicates a dominant surface-controlled contribution, while the N 1 s fitting supports the presence of pyridinic, pyrrolic and graphitic nitrogen. These results support, but do not independently quantify, the contribution of each nitrogen species.

From a practical viewpoint, algae-derived carbon is attractive because algae biomass is renewable, rapidly available and intrinsically heteroatom-containing. The optimized material also shows measurable performance in a symmetric aqueous device, which is more relevant than three-electrode capacitance alone. At the same time, several challenges remain. Natural algae composition varies with species, season, growth environment and harvesting method, which may affect ash content, nitrogen content and carbon yield. KOH activation is effective but chemically aggressive, requiring acid washing and wastewater treatment. In addition, the present aqueous device should be further tested under higher mass loading, lean electrolyte conditions and practical cell formats before scale-up can be assessed. These limitations define the next steps for translating algae-derived porous carbon from laboratory electrodes to more realistic supercapacitor devices.

## 4. Conclusions

This work prepared algae-derived hierarchical porous carbon electrodes through pre-carbonization and controlled KOH activation. The study addresses a material-design trade-off: chemical activation is required to generate ion-accessible pores, but overly severe activation can remove heteroatom functionalities and damage conductive carbon domains. By comparing temperature-, KOH-ratio- and time-dependent samples, AHPC-850 was identified as the optimized material under the tested conditions.

AHPC-850 exhibits a BET surface area of 1486 m^2^ g^−1^, a total pore volume of 0.96 cm^3^ g^−1^, a retained surface nitrogen content of 2.91 at.%, and a charge-transfer resistance of 0.41 Ω. It delivers 386 F g^−1^ at 1 A g^−1^, retains 62.4% capacitance from 1 A g^−1^ to 20 A g^−1^, and maintains 96.3% capacitance after 10,000 cycles with stable coulombic efficiency. A symmetric aqueous device provides an energy density of 24.8 Wh kg^−1^ at 250 W kg^−1^.

Overall, the results suggest that algae-derived carbon electrodes should be designed by balancing pore accessibility, nitrogen retention, wettability and electronic/ionic transport rather than by maximizing BET surface area alone. Future work should address precursor variability, greener activation routes, high-loading electrodes, operando mechanism analysis and device-level scale-up.

## Figures and Tables

**Table 1:** Measured proximate and elemental composition of the algae precursor used to rationalize its self-doping capability. Values are reported on a dry basis.

Category	Parameter	Value	Relevance to Carbon-Electrode Design
Proximate analysis	Moisture	7.8 wt.%	Determines drying requirement and mass normalization
	Volatile matter	61.5 wt.%	Controls gas evolution and carbonization shrinkage
	Fixed carbon	18.7 wt.%	Contributes to carbon framework formation
	Ash	12.0 wt.%	May influence activation and requires post-washing
Elemental analysis	C	42.6 wt.%	Primary carbon source
	H	6.3 wt.%	Volatilization during pyrolysis
	N	6.9 wt.%	Intrinsic self-doping source
	O	33.1 wt.%	Surface polarity and gas-forming reactions
	S	0.8 wt.%	Minor heteroatom contribution

**Table 2:** Experimental matrix used to optimize the AHPC synthesis.

Series	Fixed Parameters	Variable	Sample Codes	Purpose
Temperature series	char:KOH = 1:3; 1.5 h	700–900 °C	AHPC-700 to AHPC-900	Identify pore/N retention balance
KOH-dosage series	850 °C; 1.5 h	char:KOH = 1:1–1:4	AHPC-850-R1 to R4	Evaluate etching strength
Time series	850 °C; char:KOH = 1:3	0.5–2.5 h	AHPC-850-t0.5 to t2.5	Evaluate activation duration
Control series	no KOH	850 °C	AC-850	Separate carbonization from activation

**Table 3:** Synthesis-optimization results showing why AHPC-850 prepared with a char:KOH ratio of 1:3 and 1.5 h activation is selected as the optimized condition, with a BET surface-area maximum near 1500 m^2^ g^−1^.

Optimization Variable	Condition	Yield	$S_{\mathrm{BET}}$	N Content	$R_{\mathrm{ct}}$	$C_{1\;A\;g^{-1}}$
		(wt.%)	(m^2^ g^−1^)	(at.%)	(Ω)	(F g^−1^)
Temperature	700 °C	25.4	842	3.82	1.08	236
Temperature	800 °C	20.7	1218	3.34	0.72	318
Temperature	850 °C	17.9	1486	2.91	0.41	386
Temperature	900 °C	15.2	1364	2.28	0.61	342
KOH ratio	1:1	24.6	768	3.48	0.94	255
KOH ratio	1:2	21.8	1186	3.12	0.58	337
KOH ratio	1:3	17.9	1486	2.91	0.41	386
KOH ratio	1:4	13.6	1398	2.36	0.55	351
Time	0.5 h	23.1	982	3.26	0.86	286
Time	1.5 h	17.9	1486	2.91	0.41	386
Time	2.5 h	14.4	1336	2.44	0.57	348

**Table 4:** Textural, chemical and electrochemical properties of the prepared carbon samples.

Sample	*S*_*BET*_	*V*_*tot*_	N Content	$I_{D}/I_{G}$	$R_{\mathrm{ct}}$	$C_{1Ag^{-1}}$
	(m^2^ g^−1^)	(cm^3^ g^−1^)	(at.%)	–	(Ω)	(F g^−1^)
AC-850	382	0.36	4.85	1.02	1.65	181
AHPC-700	842	0.62	3.82	1.06	1.08	236
AHPC-800	1218	0.84	3.34	1.10	0.72	318
AHPC-850	1486	0.96	2.91	1.13	0.41	386
AHPC-900	1364	0.89	2.28	1.17	0.61	342

**Table 5:** N 1 s XPS fitting parameters and assignments for AHPC-850. Peak positions and FWHM values were constrained during fitting to reduce overfitting.

Nitrogen Species	Peak Position	FWHM	Relative Fraction	Residual Contribution	Electrochemical Relevance
	(eV)	(eV)	(%)	–	
Pyridinic N	398.4	1.32	42.6	RMS <2.5%	Polar edge sites; possible reversible surface redox contribution
Pyrrolic N	400.1	1.38	25.8		Improves wettability and may contribute to pseudocapacitance
Graphitic N	401.6	1.46	22.1		Promotes electronic delocalization and charge transfer
Oxidized N	404.0	1.58	9.5		Polar surface sites with limited conductivity contribution

**Table 6:** Interfacial and transport properties of algae-derived carbon electrodes.

Sample	Water Contact Angle	Electrolyte Uptake	Pellet Conductivity	Interpretation
	(Degree)	(g g^−1^)	(S m^−1^)	
AC-850	91	1.4	38	Limited porosity and sluggish wetting
AHPC-700	63	2.2	54	Partially opened pores
AHPC-800	38	3.6	71	Improved pore/wettability balance
AHPC-850	24	4.5	86	Best electrolyte accessibility and conduction
AHPC-900	31	4.2	73	Overetched framework and lower N retention

**Table 7:** Electrochemical metrics of algae-derived carbon electrodes in aqueous electrolyte. Retention is defined as $C_{20\;A\;g^{-1}}/C_{1\;A\;g^{-1}}\times 100\%$.

Sample	$C_{1Ag^{-1}}$	$C_{20Ag^{-1}}$	Rate Retention	$R_{\mathrm{ct}}$	Cycling Retention	Coulombic Efficiency
	(F g^−1^)	(F g^−1^)	(%)	(Ω)	(10,000 Cycles, %)	(10,000th Cycle, %)
AC-850	181	96	53.0	1.65	90.8	98.1
AHPC-700	236	132	55.9	1.08	92.7	98.4
AHPC-800	318	194	61.0	0.72	94.6	98.7
AHPC-850	386	241	62.4	0.41	96.3	99.2
AHPC-900	342	199	58.2	0.61	95.1	98.9

**Table 8:** Device-level performance metrics of the AHPC-850 symmetric supercapacitor. Energy and power densities are normalized to the total active mass of both electrodes.

Current/Power Condition	Cell Voltage	Device Capacitance	Energy Density	Power Density	Coulombic Efficiency
	(V)	(F g^−1^)	(Wh kg^−1^)	(W kg^−1^)	(%)
Low-power operation	1.6	69.8	24.8	250	99.1
Medium-power operation	1.6	58.4	20.8	980	99.0
High-power operation	1.6	43.7	15.5	4200	98.8
Ultra-high-power operation	1.6	32.9	11.7	8200	98.6
After 10,000 cycles	1.6	retained 95.2%	–	–	98.9

**Table 9:** Comparison with selected recent biomass-derived carbon electrodes for supercapacitors. Rate retention is reported using the current-density range stated in each cited article; therefore, the values are used for contextual comparison rather than direct ranking.

Precursor/Material	Strategy	Test System/Electrolyte	$S_{\mathrm{BET}}$ (m^2^ g^−1^)	Key Electrochemical Performance	Ref.
Defatted microalgae residue	Self-N doping and KOH activation	Three-electrode and symmetric cell; 6 M KOH	up to 3186	432 F g^−1^ at 1 A g^−1^; 46.2% retention from 0.5 to 20 A g^−1^ in the symmetric cell; 94.1% retention after 5000 cycles at 2 A g^−1^; 42.6 Wh kg^−1^ at 250 W kg^−1^	[22]
Algae-derived bio-oil residue	Self-sourced N/O/P doping and carbonate-assisted activation	Three-electrode, 6 M KOH; symmetric cell, 1 M Li_2_SO_4_	2576	452 F g^−1^ at 0.5 A g^−1^; 92.4% retention after 10,000 cycles at 10 A g^−1^; 22.6 Wh kg^−1^ at 225 W kg^−1^	[27]
Mixed biomass	In situ N/O doping by mixed-biomass activation	Three-electrode, 6 M KOH; symmetric device	3030.2	473.03 F g^−1^ at 1 A g^−1^; 49.6% retention from 0.5 to 20 A g^−1^; 95.08% retention after 10,000 cycles at 10 A g^−1^; 17.51 Wh kg^−1^ at 161.85 W kg^−1^	[23]
Reed straw/melamine	KOH activation and external N doping	Three-electrode; 6 M KOH	547.1	202.8 F g^−1^ at 1 A g^−1^; 80.9% retention from 1 to 20 A g^−1^; 96.3% retention after 5000 cycles at 20 A g^−1^	[21]
This work	Algae self-N doping and controlled KOH activation	Three-electrode, 6 M KOH; symmetric cell, 1 M Na_2_SO_4_	1486	386 F g^−1^ at 1 A g^−1^; 62.4% retention from 1 to 20 A g^−1^; 96.3% retention after 10,000 cycles at 10 A g^−1^; 24.8 Wh kg^−1^ at 250 W kg^−1^	–

## Data Availability

The data presented in this study are available within the article. Additional raw characterization and electrochemical data supporting the findings of this study are available from the corresponding authors upon reasonable request.

## Abbreviations

The following abbreviations are used in this study:

AHPC	Algae-derived hierarchical porous carbon
BET	Brunauer–Emmett–Teller
CV	Cyclic voltammetry
EIS	Electrochemical impedance spectroscopy
GCD	Galvanostatic charge–discharge
KOH	Potassium hydroxide
NLDFT	Nonlocal density functional theory
SEM	Scanning electron microscopy
TEM	Transmission electron microscopy
XPS	X-ray photoelectron spectroscopy
XRD	X-ray diffraction
